# Everyday visual demands of people with low vision: A mixed methods real-life recording study

**DOI:** 10.1167/jov.20.9.3

**Published:** 2020-09-02

**Authors:** Sandra D. Starke, Eugenie Golubova, Michael D. Crossland, James S. Wolffsohn

**Affiliations:** Aston Business School, Aston University, Birmingham, UK; Previously School of Engineering (Honorary Research Fellow), University of Birmingham, Birmingham, UK; Previously GiveVision, iCentrum, Birmingham, UK; NIHR Biomedical Research Centre for Ophthalmology at Moorfields Eye Hospital and UCL Institute of Ophthalmology, London, UK; Optometry and Vision Science, Aston University, Birmingham, UK

**Keywords:** LVA, activity inventory, wearable sight aid, sight loss, vision aid requirements

## Abstract

Research has demonstrated that low vison aids (LVAs) can have a positive impact on the functional sight of those living with sight loss. Step changes in technology are now enabling new wearable LVAs with greater potential than those available previously. For these novel devices to receive increased acceptance and therefore adoption by those with sight loss, visual task demands have to be understood more clearly in order to enable better alignment between device design and user requirements. The aim of this study was to quantify these requirements. Thirty-two participants aged 18 to 87 wore a spectacle-mounted video camera to capture and narrate all everyday situations in which they would use a “perfect” sight aid during 1 week. Captured scenes were analyzed through categorization and computational image analysis. Results showed large variation in activities and lifestyles. Participants reported no available sight aid or coping strategy for 57% of the recorded activities. Reading made up 49% of all recorded tasks, the other half comprising non-textual information. Overall, 75% of captured activities were performed ad hoc (duration of 0–5 minutes), 78% occurred indoors, 58% occurred at home, 48% were lit by natural light, 68% included the object of interest within reach, and 69% required a single focus plane only. Around half of captured objects of interest had a size of 2 degrees visual angle (2.08 logarithm of the minimum angle of resolution [logMAR]) or smaller. This study highlights the need for a sight aid that can make both textual and non-textual scenes accessible while offering flexibility to accommodate individual lifestyles.

## Introduction

Globally, approximately 216 million people live with moderate to severe visual impairment (Bourne, Flaxman, Braithwaite, Cicinelli, Das, Jonas, et al., 2017), with these numbers predicted to rise over coming decades due to an aging population (Access Economics Pty Ltd, 2009; Pezzullo, Streatfeild, Simkiss, & Shickle, 2018). Although for many people sight loss cannot be reversed or cured, low vision aids (LVAs) can have a positive impact on residual functional sight (Crossland, Starke, Imielski, Wolffsohn, & Webster, 2019; Binns, Bunce, Dickinson, Harper, Tudor-Edwards, Woodhouse, et al., 2012; Hooper, Jutai, Strong, & Russell-Minda, 2008; Peterson, Wolffsohn, Rubinstein, & Lowe, 2003; Virgili, Acosta, Bentley, Giacomelli, Allcock, & Evans, 2018), with a prominent research focus on restoring the ability to read (Virgili et al., 2018). Optical low vision aids tend to be task specific, with constraints on magnification, field of view, working distance, and ease of use: stronger optical magnifiers typically have a smaller field of view, are harder to align, and are more affected by hand movement than weaker devices. However, with the recent advent of high-resolution digital screens and miniaturization of sensing and computing devices, new wearable sight enhancement devices have become available. These devices are suitable for a broader range of activities beyond reading and overcome several challenges associated with high levels of optical magnification (Deemer et al., 2018; Ehrlich et al., 2017; Peterson, Wolffsohn, Rubinstein, & Lowe, 2003).

The opportunity of new technological developments requires that the actual visual task demands of people living with low vision have to be understood better in order to design for their specific needs through patient centric design. This would prevent falling short on user acceptance as exhibited by many current devices (Gori, Cappagli, Tonelli, Baud-Bovy, & Finocchietti, 2016). The lack of comprehensive evidence for the LVA needs and requirements of different patient groups has been highlighted through several reviews of the current low vision rehabilitation landscape (Binns et al., 2009; Binns et al., 2012; Dickinson et al., 2011; Markowitz, 2006; Markowitz, 2016). Among the few studies that do exist, the Royal National Institute for the Blind (RNIB) conducted one of the most comprehensive surveys on the self-reported impact of sight loss through the 2015 “MyVoice” study with > 1200 participants (Slade & Edwards, 2015). Similarly, the Massof inventory provides an extensive list of tasks relevant for people with low vision in the early 2000s (Massof, Hsu, Baker, Barnett, Park, Deremeik, 2005a; Massof, Hsu, Baker, Barnett, Park, Deremeik, 2005b; Massof et al., 2007). Other studies reported tasks that patients with macular degeneration struggled with most (Taylor, Hobby, Binns, & Crabb, 2016), potential use cases for LVAs (Palmer, 2005), or psychological factors for device uptake among the elderly (Horowitz, Brennan, Reinhardt, & MacMillan, 2006). Further, several studies that offer indirect clues on device requirements have examined quality of life in patients with low vision (Berdeaux, Nordmann, Colin, & Arnould, 2005; Frick et al., 2012; Lamoureux et al., 2008; Murphy et al., 2007; Tadic, Cooper, Cumberland, Lewando-Hundt, & Rahi, 2016), requirements for LVAs (Sandnes, 2016), or self-support strategies (Smallfield, Berger, Hillman, Saltzgaber, Giger, & Kaldenberg, 2017). To date, no study has systematically captured daily needs through live recordings rather than introspection.

Recording of everyday activities is a common technique applied across disciplines, such as Human Computer Interaction (HCI), Human Factors/Ergonomics and Psychology. It allows to assess behavior, interventions, and concept designs. Diary studies (Brown, Costley, Friend, & Varey, 2010; Carter & Mankoff, 2005; Karisalmi & Nieminen, 2017) utilizing electronic recording devices are popular to capture data with minimum disruption. In photo elicitation studies, participants capture imagery of activities and/or environments, commenting on them in a subsequent interview (Clark-Ibáñez, 2004; Harper, 2002; Richard & Lahman, 2015; Van House, 2006). “Ecological assessment” (Shiffman, Stone, & Hufford, 2008) or “experience sampling” (Barrett & Barrett, 2001; Christensen, Barrett, Bliss-Moreau, Lebo, & Kaschub, 2003; Intille, Kukla, & Ma, 2002) similarly capture behaviors and experiences through image recordings. Since the emergence of small wearable cameras, automated recordings of daily routines and experiences has been facilitated through devices, such as SenseCam, Autographer, Narrative, or Google Glass (Mavoa, Oliver, Kerr, Doherty, & Witten, 2013; Shipp, Skatova, Blum, & Brown, 2014; Wilson, Jones, Schofield, & Martin, 2016). These devices have been used in studies including social behavior and behavior change (Doherty et al., 2013; Fleck, 2012; Fleck & Fitzpatrick, 2009; Gemming, Doherty, Utter, Shields, & Ni Mhurchu, 2015; Mavoa et al., 2013; Wilson, Jones, Schofield, & Martin, 2016) with an associated well-established ethical framework for such recordings (Doherty et al., 2013; Kelly et al., 2013; Kienzler, 1997; Kwok, Skatova, Shipp, & Crabtree, 2015; Mok, Cornish, & Tarr, 2015; Shipp, Skatova, Blum, & Brown, 2014; Skatova et al., 2015).

The objective of this study was to develop a comprehensive list of design input requirements for future LVAs that can address real-life challenges of people with visual impairment. The aim of this study was to understand daily visual demands with regard to image content, desired activities in need of support, task characteristics, and personal priorities. The study was conducted using a mixed methods design combining self-recording of tasks through a spectacle-mounted video camera, including live narrative analyzed both qualitatively and quantitatively including computational image analysis.

## Materials and methods

### Participants

A total of 32 participants aged 18 to 87 were recruited from the Aston Low Vision Clinic and GiveVision's volunteer network. Adult (age 18 years and over) participants with a visual impairment were included in the study. All participants met the definition of visual impairment by Leat et al. (1999): best monocular or binocular visual acuity of worse than 6/7.5, horizontal visual field of < 146 degrees to Goldmann III4e targets, or contrast sensitivity worse than 1.5 log units (Leat, Legge, & Bullimore, 1999).

Participation was voluntary and participants were free to withdraw at any point. This research was conducted in compliance with the Declaration of Helsinki and Aston University's Review Board and it was granted a favorable ethical opinion and governance approval by the Aston University Ethics Committee (no. #1280). Written informed consent was obtained from participants after explanation of the nature and possible consequences of the study. At the conception stage, the study was discussed and piloted with two visually impaired volunteers and adjustments based on feedback and observations were made prior to commencing the study.

### Task

**Figure 1.**
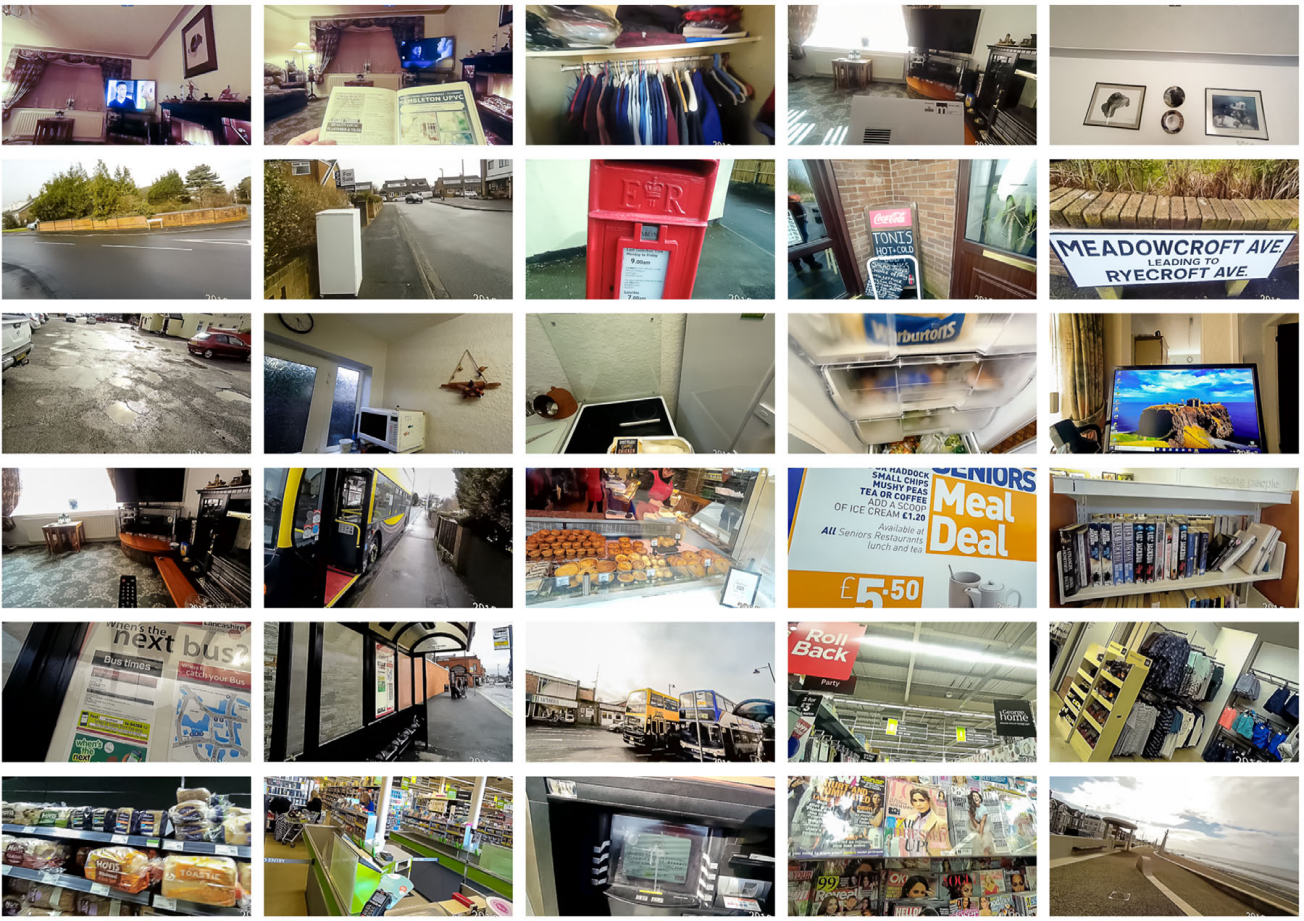
Example of scene recordings taken with the spectacle-mounted camera.

The study task was to record, for approximately 1 week, all scenarios during which the participant would use “a perfect sight aid” (see Figure 1 for an example of such recordings). Recordings were facilitated through a wearable, glasses-mounted miniature video camera. Participants were given the instructions to capture the object of interest, narrate the intended activity, and comment on the related difficulty. They were asked to record all scenarios that they struggled with, even if they had a coping strategy, such as the use of a magnifier.

Participants were equipped with recording spectacles (SunnyCam Sport; image resolution 1920 × 1080 pix, 30 fps, 120 degrees field-of-view horizontal, stereo audio recording, operation through a single button, and vibration feedback). For improved accessibility, a bumpon sticker (3M, Two Harbors, MN, USA) was affixed with superglue (Loctite, Westlake, OH, USA) to both the recording button and USB charger connectors. The device could not be used in conjunction with spectacles due to the close fit. However, none of the participants frequently wore spectacles. Participants who used yellow tinted lenses for management of light sensitivity had the device's default glasses replaced by custom yellow inserts available via the device manufacturer.

After fitting the device, a detailed practical induction on how to operate and charge it was given, verified by the participant executing set tasks, and reviewing these with the instructor. To extend battery life, participants were equipped with and trained to use a portable USB charger (Anker 2-port USB wall charger, China). Explanatory handouts for the public were provided in line with recommendations made for recording studies (Kelly et al., 2013) to move the explanatory burden from the participant to the study team. Participants were further instructed to secure verbal consent from any persons recorded in private spaces.

On return of the recording spectacles, a debrief was conducted during which participants could review recordings and answered a structured questionnaire. Results of this questionnaire will be reported separately.

### Visual assessment

During the briefing session, the participant's best corrected distance binocular visual acuity (early treatment diabetic retinopathy study [ETDRS] logarithm of the minimum angle of resolution [logMAR] chart) and contrast sensitivity (Pelli-Robson chart) were measured. Sight loss condition, onset, and demographic details were recorded.

### Data analysis

All recordings were downloaded from the recording glasses in mp4 format and stored with an anonymous identifier. Recordings were watched in real-time by the research team, extracting representative image frames for all captured scenes/subtasks, and conducting a classification of these as described below.

### Activity classification

Activities were written down as tasks as narrated by the participant. For activity, where the narrative only described a top-level task (e.g. “I am making cake”), the study team broke this task down into subtasks and used simple language. For task difficulty, the study team converted the narrative to a rating on the six-point scale of very easy, easy, average, difficult, very difficult, and unable. Repeatability analysis showed that this classification had poor repeatability between observers, and it was hence simplified to “able to complete alone” and “unable to complete alone.”

Recorded subtasks were then mapped to the 48-item Veterans Affairs Low Vision Visual Functioning Questionnaire (VA LV VFQ-48). This tool was chosen from seven validated questionnaires as it demonstrated the best fit (80%) for the captured tasks. Twelve of the VA LV VFQ-48 tasks were adapted to reflect the captured activities. An additional, bespoke task list (identified as the category ‘Bespoke Tasks’) was created specifically to capture those activities that could not be mapped to the VA LV VFQ-48.

### Scene classification

Scene classification focused on 14 characteristics related to environment, object location, and task requirements (Table 1) coded by the study team. Repeatability (see Table 1) was calculated across two authors for each of these classifiers, who independently coded the same 27 snapshots.

**Table 1.** Scene classification.

Count	Classifier	Categories	Repeatability
1	Task duration	Ad hoc (0–5 min), short (6–10 min), medium (11–30 min), long (31+ min)	72%
2	Location	Indoors, outdoors	94%
3	Familiarity	Home, public, work, school	78%
4	Light type	Artificial, natural, mixed, backlit, other	72%
5	Brightness	Low (e.g. dimly lit pub, cinema exit), medium (e.g. normally lit home environment, outside with dark clouds), bright (e.g. most days outside, brightly lit supermarket)	78%
6	Distance of object	Within reach (up to 1 m), short (up to 4 m, length of double bedroom as proxy), medium (up to 10 m, length of large coach as proxy), long (beyond 10 m to infinity)	84%
7	Focus plane	Single (no change in focus required), multiple (change required)	79%
8	Periphery required	None, side, bottom, both	79%
9	Visual scanning/search required	Yes, no	89%
10	Hands required	None, 1 hand, 2 hands	84%
11	Walking required	Yes, no	100%
12	Sight aid or coping strategy used	Yes, no	89%
13	Ability to complete alone	Yes, no	89%
14	Time of day	6–12, 12–18, 18–24, 0–6	Transcribed from video

Classifiers and their categories used to classify the captured scene content. The repeatability score between two independent assessors for each classifier is presented in the right column.

### Computational image analysis

**Figure 2.**
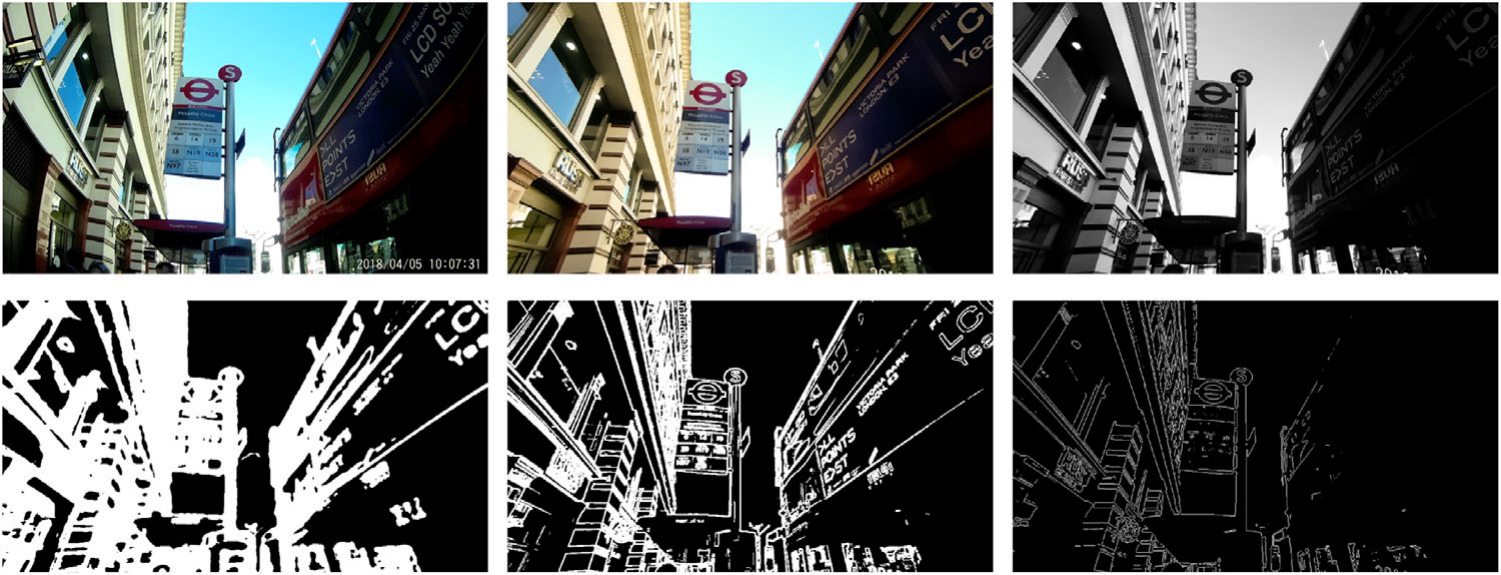
Digital image processing for the analysis of image characteristics. Top row: Left - original recording, middle - undistorted, white balanced, and de-noised image, and right - linearized greyscale image. Bottom row: Left - local entropy (texture), middle - high frequencies, and right - edges.

Exported video frames were undistorted, white-balanced, and de-noised in Lightroom 5 (Adobe Systems, Mountain View, CA, USA) and saved as 16-bit tiff images. Computerized image analysis was then conducted using Matlab 2018b (The MathWorks) with the Image Processing Toolbox through the steps described below (see Figure 2 for an illustration of the processing steps).

### Preprocessing

Recordings were preprocessed for further analysis by linearizing the sRGB color images using function “rgb2lin” and then converting the linear red, green, and blue (RGB) image to greyscale (luminance) using function “rgb2gray.” Subsequent computational image analysis was performed on this single-channel greyscale image.

### Contrast

Image contrast, as the percentage of total tonal value range captured, was calculated from the average of the 0.5% brightest (I_max_) and 0.5% darkest (I_min_) pixels for the 16-bit tiff images as

(1) $$\mathrm{Contrast}=\left(\left(I_{\max}-I_{\min}\right)/2^{16}\right)*100.$$

### Global and local entropy

Global and local entropy were calculated to characterize the texture of the image, with entropy also considered its randomness. Global entropy was computed using Matlab's “entropy” function, normalized to a 0 to 1 output space (with the boundaries of 0 – no randomness and 1 – maximum randomness). Local entropy was computed using Matlab's “entropyfilt” function (9-by-9 neighborhood) to characterize what area of the scene (A_textured_) may attract attention due to textual content. This area was calculated based on the derived entropy matrix by counting the number of pixels within the entropy matrix with a value larger than three (pix_texture,_ cut-off set experimentally as the value adequately separating texture from noise) as a proportion of all pixels (pix_all_) as

(2) $$A_{\mathrm{textured}}=100\;*\;\left(\mathrm{pi}x_{\mathrm{texture}}/\mathrm{pi}x_{\mathrm{all}}\right).$$

### High frequency content and edges

High spatial frequency content was approximated using Matlab's “rangefilt” function, which computes the tonal range (maximum – minimum luminance) for a 3-by-3 neighborhood around any given pixel. The proportion of the image containing high spatial frequency content was calculated based on the derived matrix as the area (A_highSpatialFrequency_) containing pixel values larger than 10% of the total possible range (pix_highFrequency_, threshold = 0.1*2^16^ = 6554) as a proportion of all pixels (pix_all_) as

(3) $$\;\;\;\begin{array}{c} A_{\mathrm{highSpatialFrequency}}=100\;*\left(\mathrm{pi}x_{\mathrm{highFrequency}}/\mathrm{pi}x_{\mathrm{all}}\right). \end{array}$$

Edge detection was performed through Matlab's “edge” function and the area containing edges (A_edges_) calculated as the proportion of pixels containing edges (pix_edge_) of all pixels (pix_all_) as

(4) $$\begin{array}{c} A_{\mathrm{edges}}=100\;*\left(\mathrm{pi}x_{\mathrm{edge}}/\mathrm{pi}x_{\mathrm{all}}\right). \end{array}$$

### Object size estimation

Object size (in degrees of visual angle) within the field of view was calculated for the horizontal and vertical distance, which the overall object of interest spanned within the recorded snapshot. For written content, the size of a single letter was estimated.

## Results

### Participation

Of 32 participants, 15 were women and 17 were men; 26 participants still had vision in both eyes and 6 had vision in one eye only; 16 participants had adult onset of visual impairment, 8 participants childhood onset, and 8 participants had been visually impaired from birth. Mean (SD) age of participants was 47.5 (21.9) years (range: 18 to 87 years), binocular distance acuity was 1.2 (0.3) logMAR (range: 0.36 to hand movements [HMs]), binocular contrast sensitivity was 0.8 (0.4) log units (range: 0.1 to 1.5 log units), and time since diagnosis was 22 years (16 years; range: 2 to 64 years). Initially a further four participants had joined the study. However, one participant dropped out during the study and three participants had missing recording data due to errors made by the dispensing team; these participants are not included in the *N* = 32 count participant cohort presented in the paper.

Across all recordings and participants, a total of 612 subtasks were extracted. Individual participants captured a mean (SD) of 18.9 (18.7) of these subtasks (median: 12, range: 3 to 68 per participant). Recordings varied in length from around 10 seconds to over 25 minutes; long recordings typically contained multiple tasks and subtasks as well as long un-narrated periods.

### Activity classification

Of all recorded subtasks, the most frequently used words recorded were “read” (299 of 605 words, 49%), “find” (46 words, 8%), “see” (36 words, 6%), “identify” (31 words, 5%), “bus” (29 words, 5%), “operate”(26 words, 4%), “labels” (21 words, 3%), and “TV” (20 words, 3%).

**Figure 3.**
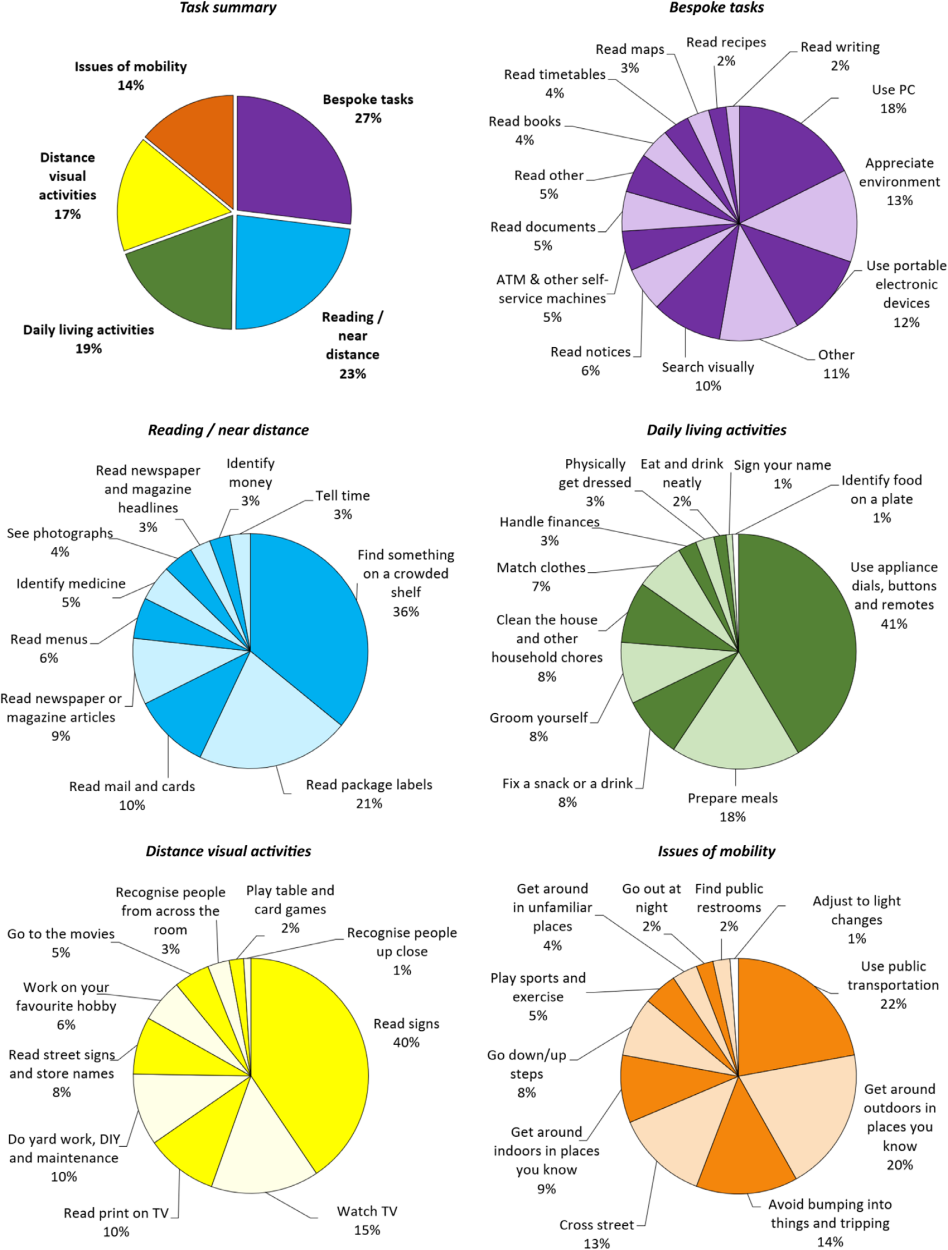
Breakdown of subtasks as mapped to the Veterans Affairs Low Vision Visual Functioning Questionnaire and bespoke tasks. Percentages are expressed relative to all tasks in a given category. For absolute values, please refer to Table 2.

Across all captured 612 scenes, the most commonly categorized subtasks fell into the “bespoke” category (27%, Figure 3). The remainder of subtasks was distributed between the four top-level categories of the VA LV VFQ-48 (see Figure 3) ranging from 14% (issues of mobility) to 23% (reading/near distance tasks). Within each of these categories, there was a small number of tasks that were substantially more frequent than the majority of others (see Figure 3).

The three most commonly reported tasks across participants were finding something on a crowded shelf, reading package labels, and using appliance dials, buttons, and remotes - 59% of participants reported each of these. Table 2 shows the ranking across all snapshots and across participants.

**Table 2.** Task rankings.

Task – all snapshots	% of all tasks	Ranking	Tasks – unique per participant	% of participants
Find something on a crowded shelf	8%	1	Find something on a crowded shelf	59%
Use appliance dials, buttons, and remotes	8%	2	Read package labels	59%
Read signs	7%	3	Use appliance dials, buttons and remotes	59%
Read package labels	5%	4	Read signs	53%
Use PC	5%	5	Watch TV	44%
Prepare meals	3%	6	Read mail and cards	38%
Appreciate environment	3%	7	Use PC	38%
Use public transportation	3%	8	Read newspaper or magazine articles	34%
Use portable electronic devices	3%	9	Use portable electronic devices	31%
Other	3%	10	Use public transportation	28%
Get around outdoors in places you know	3%	11	Appreciate environment	25%
Search visually	3%	12	Cross street	25%
Read mail and cards	2%	13	Read documents	25%
Watch TV	2%	14	Read print on TV	25%
Read newspaper or magazine articles	2%	15	Read street signs and store names	25%
Avoid bumping into things and tripping	2%	16	Search visually	25%
Cross street	2%	17	ATM and other self-service machines	22%
Read print on TV	2%	18	Get around outdoors in places you know	22%
Do yard work, DIY, and maintenance	2%	19	Read books	22%
Fix a snack or a drink	2%	20	Read menus	22%
Groom yourself	2%	21	Read other	22%
Clean the house and other household chores	2%	22	Avoid bumping into things and tripping	19%
Read notices	2%	23	Get around indoors in places you know	19%
ATM and other self-service machines	1%	24	Groom yourself	19%
Read documents	1%	25	Match clothes	19%
Read other	1%	26	Other	19%
Read menus	1%	27	Read notices	19%
Read street signs and store names	1%	28	Read timetables	19%
Match clothes	1%	29	Do yard work, DIY and maintenance	16%
Get around indoors in places you know	1%	30	Fix a snack or a drink	16%
Identify medicine	1%	31	Identify medicine	16%
Go down/up steps	1%	32	Prepare meals	16%
Read books	1%	33	Work on your favourite hobby	16%
See photographs	1%	34	Clean the house and other household chores	13%
Work on your favorite hobby	1%	35	Go down/up steps	13%
Read timetables	1%	36	Read maps	13%
Go to the movies	1%	37	Read recipes	13%
Read maps	1%	38	See photographs	13%
Read newspaper and magazine headlines	1%	39	Tell time	13%
Identify money	1%	40	Identify money	9%
Tell time	1%	41	Read newspaper and magazine headlines	9%
Play sports and exercise	1%	42	Recognize people from across the room	9%
Read recipes	1%	43	Find public restrooms	6%
Recognize people from across the room	0.5%	44	Go out at night	6%
Handle finances	0.5%	45	Play sports and exercise	6%
Physically get dressed	0.5%	46	Read writing	6%
Get around in unfamiliar places	0.5%	47	Adjust to light changes	3%
Read writing	0.5%	48	Eat and drink neatly	3%
Play table and card games	0.3%	49	Get around in unfamiliar places	3%
Eat and drink neatly	0.3%	50	Go to the movies	3%
Go out at night	0.3%	51	Handle finances	3%
Find public restrooms	0.3%	52	Identify food on a plate	3%
Recognize people up close	0.2%	53	Physically get dressed	3%
Sign your name	0.2%	54	Play table and card games	3%
Identify food on a plate	0.2%	55	Recognize people up close	3%
Adjust to light changes	0.2%	56	Sign your name	3%

Ranking of captured tasks. Left – ranking by the overall frequency of task occurrence relative to all captured tasks across all participants. For example, a single participant may report three activities related to ‘find something on a crowded shelf’: in a supermarket, in the kitchen and at work. This would add a count of three to the total frequency. Right – ranking by the frequency of task occurrence between individual participants. The example participant above would add a count of one to this frequency, as he/she reported it.

Please note: “Handle finances” and “ATM” were coded separately: finances may include tasks, such as writing a check, making transactions, or checking an account balance online, all usually privately at home. Using an ATM is focused on operating buttons, touch screens, and reading screen content, usually in the public.

### Scene classification

Participants reported use of a sight aid or other coping strategy (such as help from others or touch) for 43% of recorded scenes, and no coping strategy for the remaining 57%. Participants were able to complete 71% of recorded scenes alone, the remaining 29% could not be completed at all or only with the help from others (examples were retrieving money from a cash point, crossing the road, choosing the correct bus, or selecting items while shopping).

**Figure 4.**
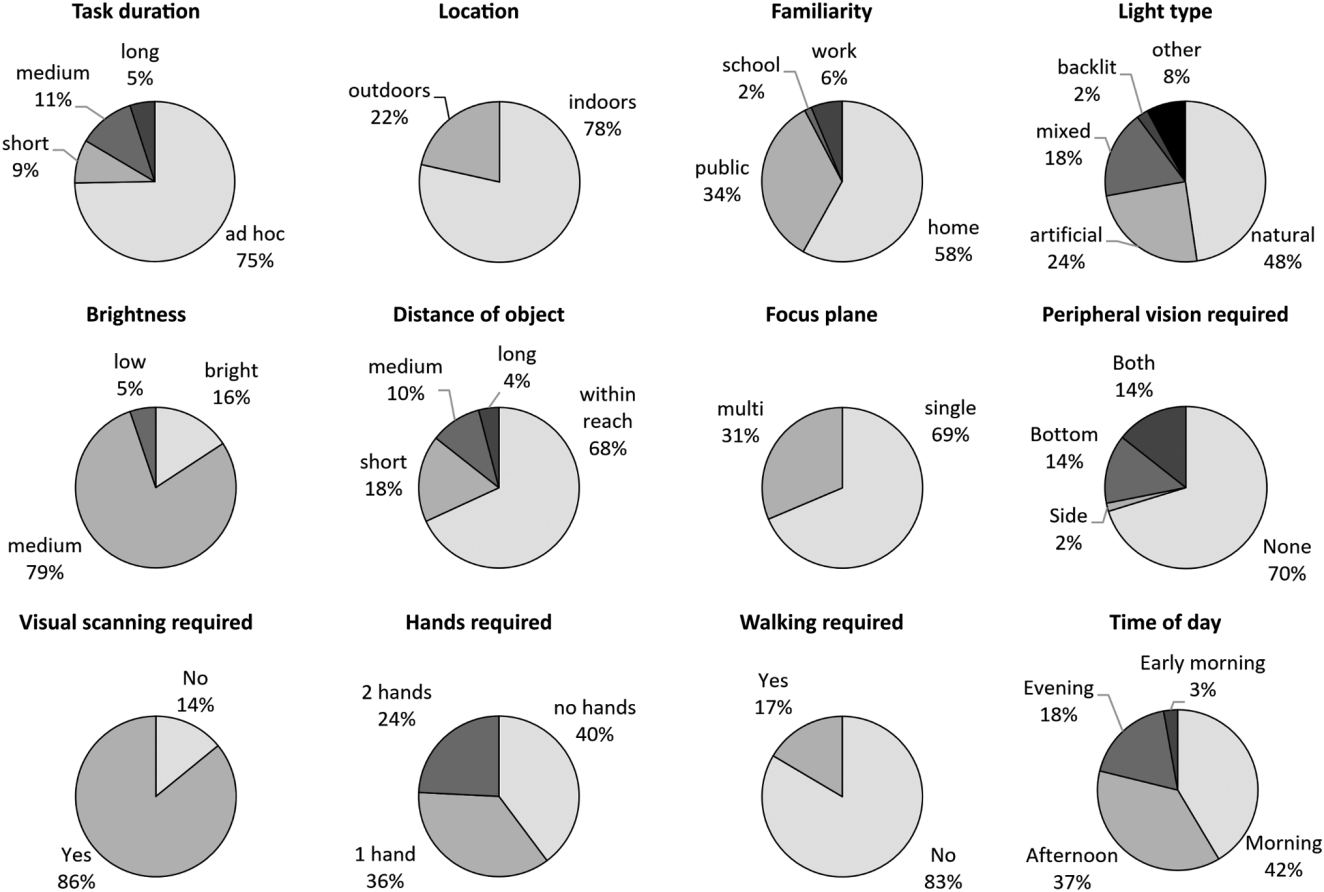
Scene characteristics of the captured recordings with regards to task context, lighting, visual, and focus requirements as well as practical requirements.

Classification of recorded scenes (Figure 4) showed that 75% of activities were performed ad hoc (duration of 0–5 minutes), 78% occurred indoors, and 58% occurred at home. Of all scenes, 48% were lit by natural light, 68% included the object of interest within reach, and 69% required a single focus plane only. To perform activities, in 60% of scenes, one or two hands were required, and in 17% walking was required. The majority of activities occurred during the morning (6:00–12:00, 42%) and afternoon (12:00–18:00, 37%).

### Computational image analysis

**Figure 5.**
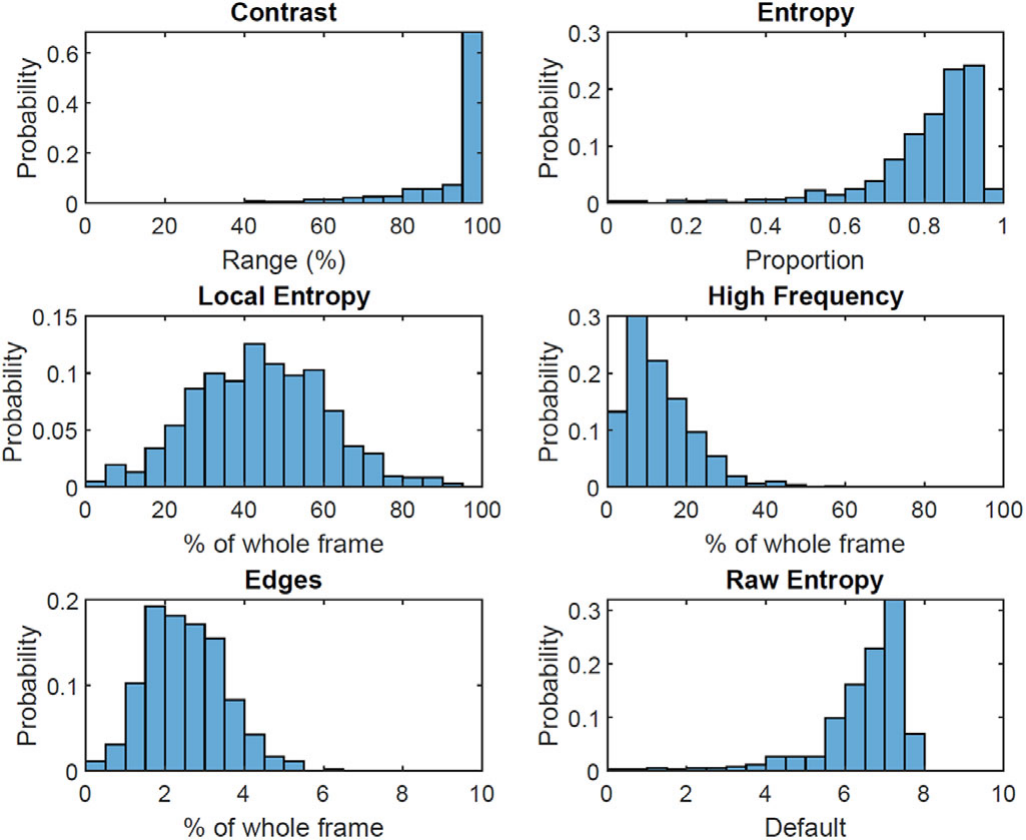
Image characteristics of all captured scenes as quantified through computational image analysis. The probability (0–1 scale) refers to the proportion of images with a given characteristic.

Across the 612 snapshots, computational image analysis (Figure 5) showed that the mean (SD) contrast range was 93.1% (12.3%) with a range from 28.5% to 100%, entropy was 0.8 (0.1) out of 1.0 with a range from 0 to 1, local entropy reflecting notable texture covered 44.0% (16.6%) of the image with a range from 1% to 92.4%, high frequency content covered 13.2% (8.5%) of the image with a range from 0.2% to 58.9%, and edges covered 2.5% (1.0%) of the image with a range from 0.2% to 6.1%.

### Object size estimation

**Figure 6.**
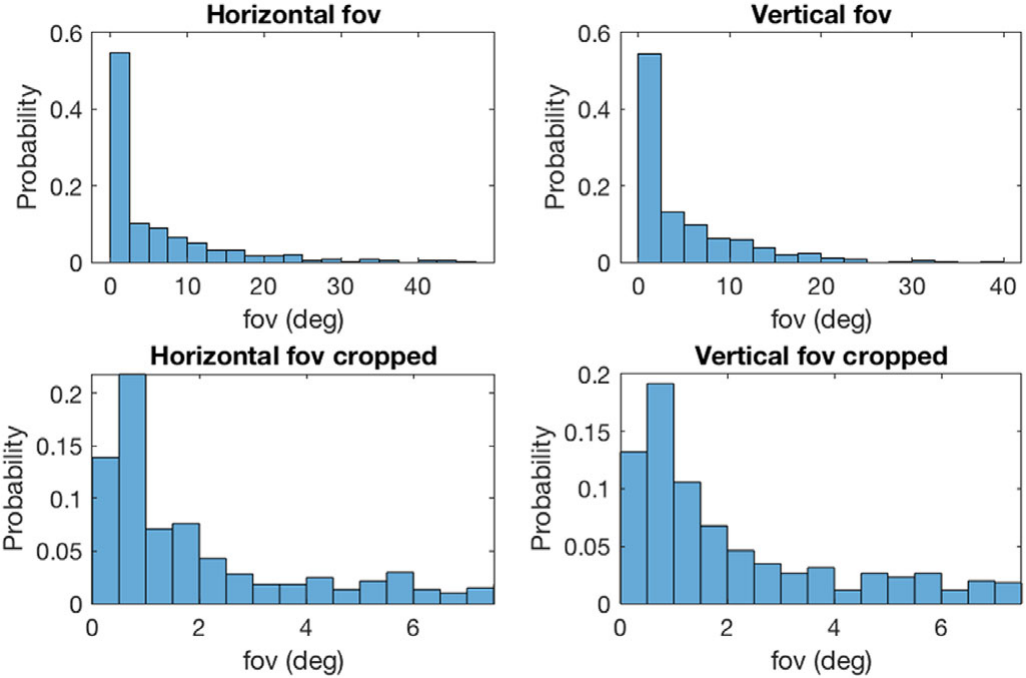
Object of interest sizes (in field of view fov) across all participants and captured objects. Note that the size relates to the object as a whole, not the textures contained within (e.g. jar of jam). For textual information, the size of a single letter was approximated.

Objects of interest had a mean (SD) size of 5.6 (7.7) degree visual angle horizontally with a range from 0.1 to 45.4 degrees (Figure 6). The logMAR equivalent of this is 2.53 (2.66) logMAR, ranging from 0.78 logMAR to 3.44 logMAR. Outcomes for vertical angles were similar at 4.7 degrees (5.8 degrees) visual angle vertically with a range from 0.2 to 37.8 degrees. The logMAR equivalent of this is 2.45 logMAR (2.54 logMAR), ranging from 1.08 logMAR to 3.36 logMAR. All reported measures do not consider texture within the object of interest, solely the dimensions of its general size. The distribution was highly skewed toward small angles. Across all snapshots, 50.4% of objects of interest had a size of < 2.0 degrees visual angle (2.08 logMAR) horizontally and 49.8% had < 2.0 degrees vertically.

## Discussion

### General summary

This study quantified the breadth of LVA requirements from both the behavioral and technical perspective. The activities recorded here compared well with previous work (Palmer, 2005; Royal National Institute for the Blind, 2015; Taylor, Hobby, Binns, & Crabb, 2016; see also Table 3), albeit with some variation in the detail of descriptions by authors and priority ranking of reported activities by participants. Most importantly, we found that activities reported in this study were not fully captured by any of the existing inventories. This highlights the need to update inventories to include daily activities which are relevant now, because behaviors, hobbies, and technology change constantly (i.e. the use of smartphones, tablets, laptops, and/or PCs is now common across age groups). One of the largest activity inventories in the field identified 337 tasks and ordered them by difficulty based on 600 participants (Massof et al., 2005), with good overlap with the tasks reported in the present study. Similar to our large number of specific activities reported by few individuals each, a smaller 2005 study (Palmer, 2005) with 25 participants (most aged 75+ years) found many specific activities reported by relatively few people, too.

**Table 3.** 

Present study	Study 1(RNIB My Voice, 2015)	Study 2(Taylor et al., 2016)	Study 3(Palmer, 2005) *N* = 25
Find something on a crowded shelf (59%)	Preparing meals (majority)	Meal preparation, shopping	Shopping (*N* = 9), baking and cooking (*N* = 8)
Read package labels (59%)	Information on medication or food packaging quite difficult or impossible to read (90%)	Reading	
Read signs (53%)	Read written information (half, always, or frequently)		–
Read mail and cards (38%)			Reading my own mail (*N* = 7)
Read newspaper or magazine articles (34%)			Reading books (*N* = 16), reading the paper (*N* = 8)
Read documents (25%)			
Read print on TV (25%)			–
Read street signs and store names (25%)			–
Use appliance dials, buttons, and remotes (59%)	Setting heating controls (majority)	–	–
Watch TV (44%)	–	Watching TV	Watching TV (*N* = 10)
Use PC (38%)	–	Computer use	–
Use portable electronic devices (31%)	–	–	–
Use public transportation (28%)	Restricted making journeys and/or getting out of the house without help (almost half)	–	Doing things/going places on their own (*N* = 12), seeing bus numbers (*N* = 7)
Cross the street (25%)		–	
Appreciate environment (25%)	–	Perception of scenes	–
Search visually (25%)	–	–	–
*Captured through other categories*	Personal care (majority)	Self-care	–
*Captured through other categories*	Choosing the right clothing (majority)	–	–
–	Restricted in the activities that they were able to take part in (half)	–	Knitting, sewing, tapestry, crochet (*N* = 9), gardening/weeding/ cutting grass (*N* = 7)
–	Would like to do more physical activity (two-thirds)	–	–
–	–	Face recognition	–
*Captured through other categories*	–	Cleaning	–
–	–	–	Being independent (*N* = 17)
*Captured through other categories*	–	–	Travelling/going abroad (*N* = 10)

Tasks reported in the present study compared to three reference studies from the literature. This table maps tasks between different studies to illustrate similarities, differences in fine-grained descriptors, and task absence between different pieces of work. It illustrates that no one single study captured all potential needs reported by people with sight loss and that differences in descriptors may lead to varying classifications. Where the present study used differing descriptors compared to studies presented here, this is highlighted as “captured through other categories.”

Overall, our results illustrate that LVAs need to consider individual lifestyles and hobbies in order to be relevant for people living with low vision. A paradigm shift away from working toward satisfying “average” users and/or focusing on single tasks and instead flexibly designing toward the individual needs of diverse groups is here both the opportunity and challenge for future device developers. We have identified many diverse tasks that people with visual impairment need help with and accept that it may not be feasible for one device to help with all tasks. When moving ahead, the factors of task frequency, importance, and difficulty should be carefully explored further: a frequent task may not be important or difficult; an important task may not be frequent; and a difficult task may not be important. Our hope is that this study is seen as a starting point for much needed further research into the needs of potential subgroups.

### What everyday activities require support through LVAs?

In this study, reading text in a vast variety of contexts comprised almost half of all recorded scenes. The other half, however, contained non-textual information, highlighting the need to make accessible scenes other than text through LVAs. Comparing our findings to the independent study outcomes above, it is apparent that people with low vision require an LVA for a very varied task inventory, including indoor and outdoor tasks, recognizing many different objects, reading many different types of information presented in numerous ways at different distances, as well as recreational and professional tasks, and tasks in both familiar and unfamiliar/public environments. In developing LVAs, designers have to embrace that different people lead different lives in which different activities can make a crucial difference. Rather than developing an LVA that only supports few very common tasks, it would hence be beneficial to develop for as broad a task range as possible to maximize uptake. It remains uncertain which factors are most important when predicting device uptake, where there is, for example, disagreement between studies on the predictive power of use frequency for the perceived benefit (Lorenzini & Wittich, 2019). Ensuring that LVA design maps onto people's individual lifestyles may be a key factor leading to uptake.

This study showed that almost a third (29%) of desired activities could not be completed by participants alone. Similarly, participants did not report having a coping strategy for more than half (57%) of recorded sub-tasks. This illustrates the loss of independence that sight loss currently causes. Similarly, the 2015 RNIB “My Voice” study (Royal National Institute for the Blind, 2015) found that the majority of their over 1200 participants required help in the home, whilst two-thirds of working age people and one-third of retirees had recently experienced collisions with obstacles on the pavement (one in three incidents leading to injury). It is important to note that the inability to complete individual subtasks, such as operating dials on a cooker, can prevent people with low vision completing whole activities, such as meal preparation. Other such examples include inability to access information about busses preventing independent use of public transport or inability to cross a road preventing leaving the house. Developing LVAs that support these subtasks may open up vastly improved independence. Future work should establish such critical subtasks presenting barriers to general activities of daily living and focus on making these accessible.

### What do the characteristics of recorded scenes tell us about LVA design requirements?

Our computational image analysis showed that modern LVAs have to accommodate a broad range of scenes with sometimes highly variable characteristics. Analysis of image contrast showed that the majority of scenes use the full contrast range, hence requiring technology with a large dynamic range to capture all tonal values in mostly high contrast scenes of everyday life. A move to high dynamic range (HDR) processing might prove beneficial here. Brightness varied largely between outdoor activities and indoor activities; whereas we classified the majority of scenes as having “medium” brightness (e.g. normally lit home environment and cloudy day outside), any modern LVA will have to accommodate the extremes in order to offer support throughout the day. Based on recordings, we estimate that luminance values ranging from 20 cd/m^2^ (scene at sunrise or sunset) to 5000 cd/m^2^ (scene in full sunlight) should be reproduced without noticeable image artifacts, such as clipping (bright regions) or noise (dark regions), with optimal performance at around 700 cd/m^2^ (scene on an overcast day).

The vast majority of scenes (75%) fell into the “ad hoc” category, with an expected duration of up to 5 minutes. This requires LVAs with a quick start-up time and almost immediate access and responsiveness, as otherwise the effort and wait of engaging the LVA may prevent people from utilizing it. It is important to note that some activities, such as cooking, may require a succession of such ad hoc tasks (e.g. reading a recipe, finding ingredients, assembling a kitchen tool, and setting oven dials). The user may wish to wear the sight aid continuously for such tasks or use it only where needed, and this should be facilitated through the design. Equally, tasks such as watching TV or reading, require an LVA that is comfortable and safe to wear for hours.

Based on our scene classification results, modern LVAs should accommodate quickly changing focus planes and object distances, although three-quarters of tasks required a single focus plane for an object within reach. LVAs would benefit from allowing peripheral vision, especially for the bottom/lower periphery, and should allow for visual scanning, utilization of hands while using the LVA, and ideally facilitate walking. Given that just over half (58%) of activities were performed at home, design acceptability in public spaces, work environments, and school is of importance. Finally, we found that the size of the attended object of interest was typically less than around 2 degrees visual angle horizontally and vertically. This requires a substantial magnification factor and related requirements, such as image quality and stability, which will further scale with the user's acuity.

### Study limitations

In this study, a number of approximations had to be made given technical constraints. Calculation of contrast was relative to the system's dynamic range and exposure adjustments so they do not represent an absolute contrast estimate. Calculations of texture and areas with high spatial frequencies depended on set thresholds and should be regarded as approximations. Estimates of object size are based on the camera's field of view and measured object size in pixels, which are subject to measurement error especially for small objects.

Comprehensive briefing sessions were conducted to ensure participant compliance (practical exercises, review of recorded examples, and verification of the ability to control the recording spectacles independently). In addition, large-print instructions were provided for use and a technical helpline offered. Although this led to all participants capturing recordings, the duration and style of recordings still varied noticeably between participants. Future studies should plan for such variation and review ways how to reduce it further. To an extent, we believe that the difference in the number of recordings between participants reflects individual lifestyles. However, some participants may have recorded less due to feeling uncomfortable recording in public, etc. This should be taken into consideration when interpreting our results.

As part of this study, we did not specifically enquire about compliance. One possible explanation for 58% of recordings taking place at home could be forgetting to wear the device when going out or not wanting to be seen wearing the device in public. Another could be that 58% of activities that participants believe they would use a “perfect sight aid” for genuinely do take place in the home, giving a level of uncertainty regarding the location of device usage when interpreting results. An interesting question is whether, even if outdoor use was under-represented due to forgetting to take the device along or not wanting to be seen with it, the same bias would also apply to a real device. Designing for increased uptake outside the home would then become an additional behavior change and design challenge.
